# Metabolic engineering of *Corynebacterium glutamicum* aimed at alternative carbon sources and new products

**DOI:** 10.5936/csbj.201210004

**Published:** 2012-10-30

**Authors:** Ahmed Zahoor, Steffen N. Lindner, Volker F. Wendisch

**Affiliations:** a Genetics of Prokaryotes, Faculty of Biology & CeBiTec, University of Bielefeld, 33615 Bielefeld, Germany

**Keywords:** amino acids, organic acids, diamines, hemicellulose, glycerol

## Abstract

*Corynebacterium glutamicum* is well known as the amino acid-producing workhorse of fermentation industry, being used for multi-million-ton scale production of glutamate and lysine for more than 60 years. However, it is only recently that extensive research has focused on engineering it beyond the scope of amino acids. Meanwhile, a variety of corynebacterial strains allows access to alternative carbon sources and/or allows production of a wide range of industrially relevant compounds. Some of these efforts set new standards in terms of titers and productivities achieved whereas others represent a proof-of-principle. These achievements manifest the position of *C. glutamicum* as an important industrial microorganism with capabilities far beyond the traditional amino acid production. In this review we focus on the state of the art of metabolic engineering of *C. glutamicum* for utilization of alternative carbon sources, (e.g. coming from wastes and unprocessed sources), and construction of *C. glutamicum* strains for production of new products such as diamines, organic acids and alcohols

## 1. Introduction

Industrial biotechnology is paving the way for a bio-based economy, which is proposed to complement and, in the long term, replace petro-based economy. Microorganisms are central to this technology and carry out the production reactions whether fermentation, whole-cell biocatalysis or enzymatic reactions. Although microbial processes have been known and used since many thousands years, it is the recombinant DNA technology and more recently the application of ‘omics’ technologies that allowed scientists to rationally engineer these microbial factories as never before.

*Corynebacterium glutamicum* is a Gram-positive bacterium and is referred to as the industrial workhorse for amino acid production. It was first isolated in 1950s from Japanese soil during a quest for natural L-glutamate producers [1]. Since then it has been thoroughly investigated and used as a generally-regarded-as-safe (GRAS) organism in fermentation industry for more than 50 years. Today it is used for the annual production of 2,160,000 tons of L-glutamate [2] and 1,480,000 tons of L-lysine [3]. After genetic engineering tools were developed, *C. glutamicum* genome was sequenced and made publically available in 2003 [4, 5] and omics technologies such as transcriptome studies evolved [6]. These milestones marked the beginning of extensive research efforts to metabolically engineer *C. glutamicum*, initially for L-glutamate and L-lysine production and later on for the production of a variety of products such as organic acids, alcohols or diamines. Similarly, use of alternative carbon sources, that unlike the traditional sugars sucrose, glucose and fructose, do not have competing applications in food industry, became possible by metabolic engineering.

The following pages are intended to provide the reader with a glimpse into metabolic engineering of *C. glutamicum* for (i) utilization of alternative carbon sources and (ii) formation of non-traditional products. Metabolic engineering approaches for L-glutamate and L-lysine production have been reviewed and monographed extensively [7–12], and therefore will not be a part of this review.

## II. Metabolic engineering for carbon source utilization

### II.1 Optimization of selected endogenous substrate utilization pathways

*C. glutamicum* can utilize many carbon sources for its growth and energy supply: monosaccharides such as glucose, fructose, and ribose; disaccharides such as sucrose, mannose, and maltose; alcohols such as inositol or ethanol and organic acids such as pyruvic acid, propionic acid, lactic acid, acetic acid, and gluconic acid, but also some amino acids such as L-glutamate and L-glutamine [13, 14]. Production processes with *C. glutamicum* are carried out utilizing sugars derived from starch (glucose) or molasses (sucrose and fructose) [8, 12]. Use of molasses dominates in European, South American and Asian production plants, whereas starch hydrolysates are favored in North America [12]. In *C. glutamicum* these sugars are substrates of the phosphotransferase system (PTS), which phosphorylates its substrate by utilizing phosphoenolpyruvate [15].

#### II.1.1 Glucose

The utilization of glucose via the PTS was increased by constitutive expression of the glucose specific EII subunit gene ptsG [16]. Furthermore, a PTS-independent glucose utilization pathway, which transports glucose into the cell and then phosphorylates it using ATP and/or polyphosphate dependent glucokinases [17, 18] was shown to be present in *C. glutamicum*. When glucose was solely utilized via this system, increased L-lysine production was observed, probably due to a higher availability of the L-lysine precursor phosphoenolpyruvate [19, 20]. This alternative pathway might be favorable for a variety of production processes [21].

#### II.1.2 Lactic acid

Lactic acid is not only the predominant byproduct of *C. glutamicum* during growth with glucose under oxygen limiting conditions, but also an interesting carbon source taking its abundance in silages into account. *C. glutamicum* possesses two lactate dehydrogenases for the utilization of lactic acids, one specific for L-and one for D-lactic acid oxidization to pyruvate [22, 23]. The quinone-dependent L-lactate dehydrogenase gene (*lldD*) is sufficiently expressed for fast growth with L-lactic acid whereas D-lactate dehydrogenase gene (*dld*) needs to be overexpressed for efficient D-lactic acid utilization [22–24]. Further strain optimization by overexpression of either malic enzyme or pyruvate carboxylase genes enabled L-lysine production from lactic acid [24]. The recent observation that overexpression of the lactic acid forming NAD-dependent lactate dehydrogenase gene (*ldhA*) rescued an *lldD* knockout strain [25] indicates that *ldhA* overexpression might prove beneficial to accelerate lactate catabolism.

### II.2 Establishing access to alternative carbon sources

Several cheaply available and therefore interesting carbon sources cannot be utilized by *C. glutamicum*, either because the whole or part of the necessary genetic setup is missing or a sufficient level of gene expression is not present. Thus, the construction of recombinant strains towards utilization of further carbon sources is an important part of the metabolic engineering of this bacterium.

#### II.2.1 Starch

Glucose derived from starch hydrolysates is used in industrial amino acid production processes. In first engineering approaches towards direct utilization of starch the α-amylase gene from *Streptomyces griseus* was expressed in *C. glutamicum* and the enzyme was excreted into the medium [26]. The recombinant strain was able to grow with starch even faster as compared to glucose. However growth was only achieved when small amounts of glucose were present, and some starch degradation product remained in the medium [26]. Expression of the *Streptococcus bovis* α-amylase gene together with *Bacillus subtilis pgsA* to produce a surface displayed fusion protein, enabled *C. glutamicum* for the utilization of starch as sole source of carbon [27]. Starch-based production of L-glutamate [28], L-lysine [26, 27, 29], and cadaverine [30] has been reported and in the case of L-lysine, higher L-lysine production yields were reached with starch as compared to glucose [27].

#### II.2.2 Lactose and Galactose

Utilization of the whey sugars lactose and galactose has been engineered in *C. glutamicum*. Lactose utilization was achieved by heterologous expression of the *lac* genes from *Escherichia coli* [31]. As lactose was cleaved into its monomeric compounds glucose and galactose by β-galactosidase, *C. glutamicum* was able to utilize liberated glucose but not the galactose component of lactose [31]. Combining heterologous expression of the lactose permease and β-galactosidase genes from *Lactobacillus delbrueckii* with expression of *Lactococcus lactis* subsp. cremoris MG1363 genes for aldose^-1^-epimerase, galactokinase, UDP-glucose-I-P-uridylyltransferase, and UDP-galactose-4-epimerase allowed the use of both the glucose and galactose components of lactose [32]. Whey-based L-lysine production was possible, but growth from the whey components was slower as compared to that on glucose [32].

#### II.2.3 Arabinose

Arabinose is one of the main compounds of lignocellulosic wastes, which accrue from e.g. agricultural or forestry productions. Three enzymatic reactions are necessary for the conversion of arabinose to the pentose phosphate pathway intermediate xylulose-5-phosphate: first arabinose isomerase (AraA) converts arabinose to ribulose, second ribulokinase (AraB) phosphorylates ribulose to ribulose-5-phosphate, which finally is converted to xylulose-5-phosphate by ribulose-5-phosphate 4-epimerase (AraD) [33]. Expression of the arabinose transporter gene *araE* from *C. glutamicum* ATCC 31831 allowed utilization of at low concentrations of arabinose [34]. Arabinose has been used as a carbon source for the production of organic acids [34] as well as for the production of the amino acids L-glutamate, L-lysine, L-ornithine and L-arginine [35].

#### II.2.4 Xylose

As arabinose, the pentose sugar xylose is a component of lignocellulosic wastes. To funnel xylose into the pentose phosphate pathway, xylose has to be converted to xylulose and then phosphorylated to xylulose-5-phosphate. As *C. glutamicum* possesses a gene encoding a xylulokinase (*xylB*), the expression of the xylose isomerase gene (xylA) from *E. coli* was sufficient to enable growth from xylose as sole carbon source, but the additional expression of *E. coli xylB* accelerated growth of the recombinant *C. glutamicum* strain [36]. When optimizing arabinose utilization the arabinose transporter gene *araE* from *C. glutamicum* ATCC 31831 was expressed in the recombinant *C. glutamicum* xylose utilization strain, resulting in an increased xylose consumption rate and growth rate [34]. To prove its biotechnological usability, anaerobic production of succinic and lactic acid [36], as well as production of cadaverine [37] and the amino acids L-glutamate and L-lysine [38] from xylose was demonstrated.

#### II.2.5 Cellobiose

Cellobiose is contained in hydrolysates of cellulosic biomass. A *C. glutamicum* R mutant gained the ability to utilize cellobiose. This ability was due to mutations in the phosphotransferase permease subunit gene bglF, which is specific for the transport of the β-glucosides arbutin and salicin. Two mutations were published, both at the same position (317), resulting in amino acid exchanges from valine to alanine or methionine [39]. For the utilization of cellobiose also the phospho-β-glucosidase is needed (bglA). Both of these genes are present in *C. glutamicum* R, but are absent from others. Under oxygen deprivation conditions mixtures of cellobiose, glucose and xylose could be used for the production of lactic and succinic acid [34, 40].

#### II.2.6 Dicarboxylic acids

Fast utilization of the dicarboxylic acids namely succinic, fumaric, and malic acid (intermediates of the tricarboxylic acid cycle) was observed only in spontaneous mutants, which showed increased expression levels of genes encoding proton-or sodium-dependent transporters DctA and DccT, respectively [41, 42]. Increased expression was due to promoter-up mutations in the promoter regions of the genes. Overexpression of the corresponding genes *dctA* or *dccT* (*dcsT* in *C. glutamicum* R) resulted in fast growth with succinic, fumaric, and malic acid [41–43].

#### II.2.7 Glycerol

During the transesterfication process of oils and fats for biodiesel production, glycerol remains as a stoichiometric byproduct, with a yield of 100 kg per ton. Glycerol, previously an interesting product, also of biotechnology, became a waste material as the biodiesel industry arose. *C. glutamicum*, which cannot utilize glycerol naturally, was engineered for glycerol utilization by heterologous expression of the *E. coli* aerobic glycerol utilization genes encoding for a glycerol facilitator, glycerol kinase, and glycerol-3-phosphate dehydrogenase [44]. The plasmid borne expression of these genes enabled *C. glutamicum* for fast growth with glycerol as sole carbon source. When compared to glucose similar L-glutamate and L-lysine production properties were reached when using glycerol [44]. Using the same plasmid recently efficient succinic acid production from glycerol could be achieved [45].

#### II.2.8 Coutilization

Coutilization of substrates is a typical characteristic of *C. glutamicum* [14], and has been studied in detail with respect to the co-utilization of acetate and glucose [46, 47]. Coutilization is advantageous in biotechnological applications, but it is absent in other hosts e.g. *E. coli* [48] or *B. subtilis* [49], which show successive utilization of substrates, carbon catabolite repression, and diauxic growth. But, no rule without exceptions: *C. glutamicum* does utilize glucose before L-glutamic acid or ethanol, and acetate before ethanol [50, 51].

Moreover, when additional carbon utilization pathways are introduced into *C. glutamicum* coutilization is pertinent and has been shown for utilization of glycerol with glucose [44], lignocellulose compound xylose with glucose [36], arabinose with glucose [35], xylose and arabinose with glucose [38], and cellobiose together with xylose and glucose [34].

## III. Metabolic engineering of *C. glutamicum* for product formation

This section summarizes the wide array of metabolic products for which *C. glutamicum* has been engineered. The majority of these products are amino acids or organic acids but diamines, alcohols and other products of industrial relevance are being continuously added to this repertoire. As mentioned earlier, metabolic engineering of L-glutamate and L-lysine will not be a part of this review because they have already been extensively reviewed [7–12]. In many cases, the strategy for product metabolic engineering has evolved over time, making use of results from ongoing research in order to overcome the bottlenecks identified and to finally get the most promising strain. All pathways and their components used for the engineering of different products discussed in this section are drawn in Figure 2 and hence a reference to the same figure will not be made while discussing each product. Readers are also recommended to refer to Table 1 of another review [52] for a summary of production parameters of different *C. glutamicum* production strains.

**Figure 1:**
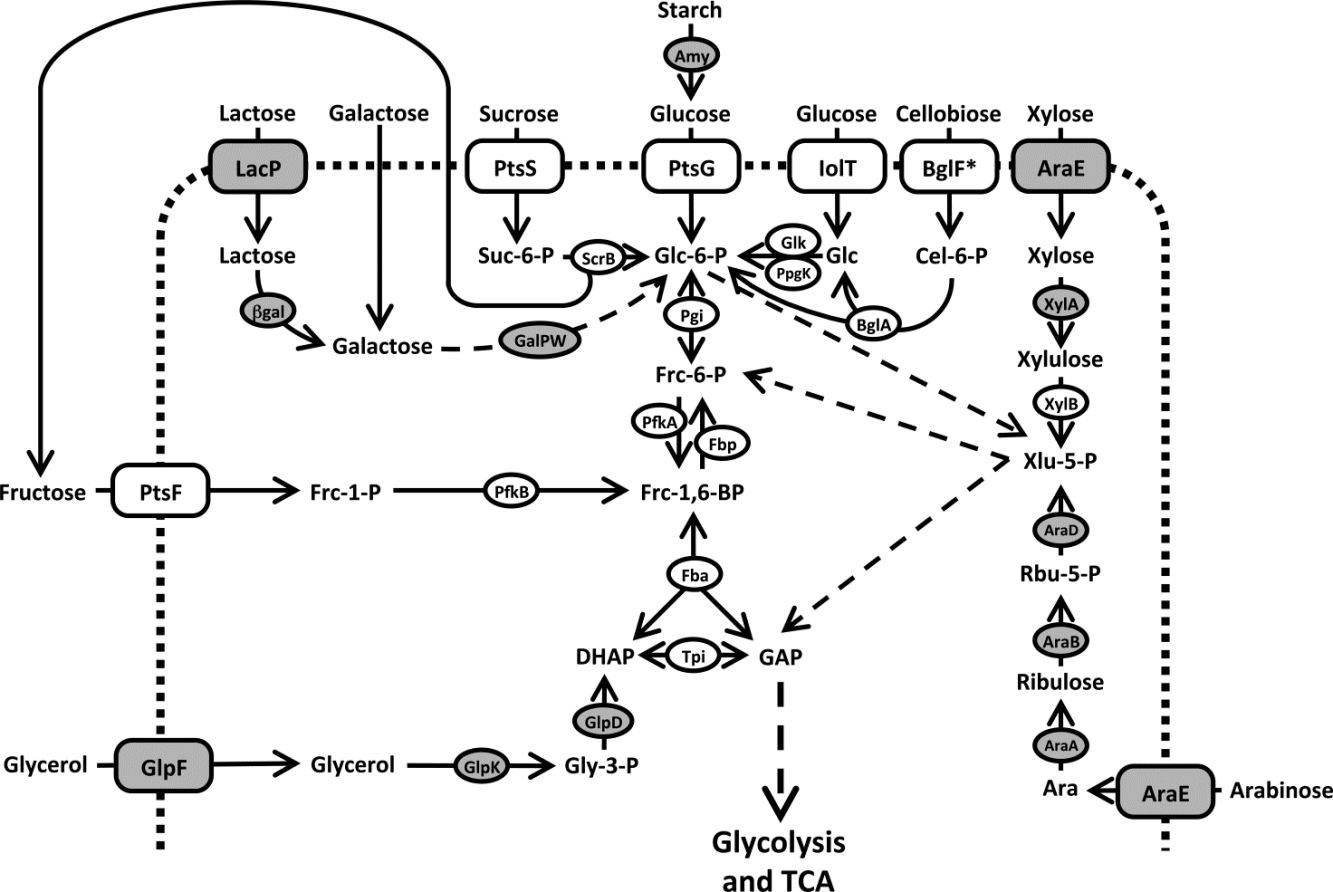
**Carbon source utilization in *C. glutamicum***. Open boxes: *C. glutamicum* enzymes, gray shaded boxes: enzymes from other organisms produced in *C. glutamicum* by heterologous expression. Abbreviations: Ara, arabinose; AraA, arabinose isomerase; AraB, ribulokinase; AraD, ribulose 5-phosphate 4-epimerase; AraE, arabinose transporter; βgal, β-galactosidase; BglA, phospho-β-glucosidase; BglF*, cellobiose specific PTS; Cel-6-P, cellobiose-6-phosphate; DHAP, dihydroxyacetonephosphate; Fba, fructose-bisphosphate aldolase; Fbp, fructose-bisphosphatase; Frc-1-,6-BP, fructose-1,6-bisphosphate; Frc-6-P, fructose-6-phosphate; Frc-1-P, fructose-1-phosphate; GalPW, galactose pathway; GAP, glyceraldehyde-3-phosphate; Glc-6-P, glucose-6-phosphate; Glc, glucose; Glk, ATP dependent glucokinase; GlpD, glycerol-3-phosphate dehydrogenase; GlpF, glycerol facilitator; GlpK, glycerol kinase; Gly-3-P, glycerol-3-phosphate; IolT, inositol transporter, also accepting glucose; LacP, lactose permease; PfkA, 6-phosphofructokinase; PfkB, 1-phosphofructokinase; PpgK, polyphosphate dependent glucokinase; PtsF, fructose specific PTS; PtsG, glucose specific PTS; PtsS, sucrose specific PTS; Rbu-5-P, ribulose-5-phosphate; ScrB, sucrose-6-phosphate hydrolase; Suc-6-P, sucrose-6-phosphate; Tpi, triosephosphate isomerase; Xlu-5-P, xylulose-5-phosphate; XylA, xylose isomerase; XylB, xylulokinase.

**Figure 2:**
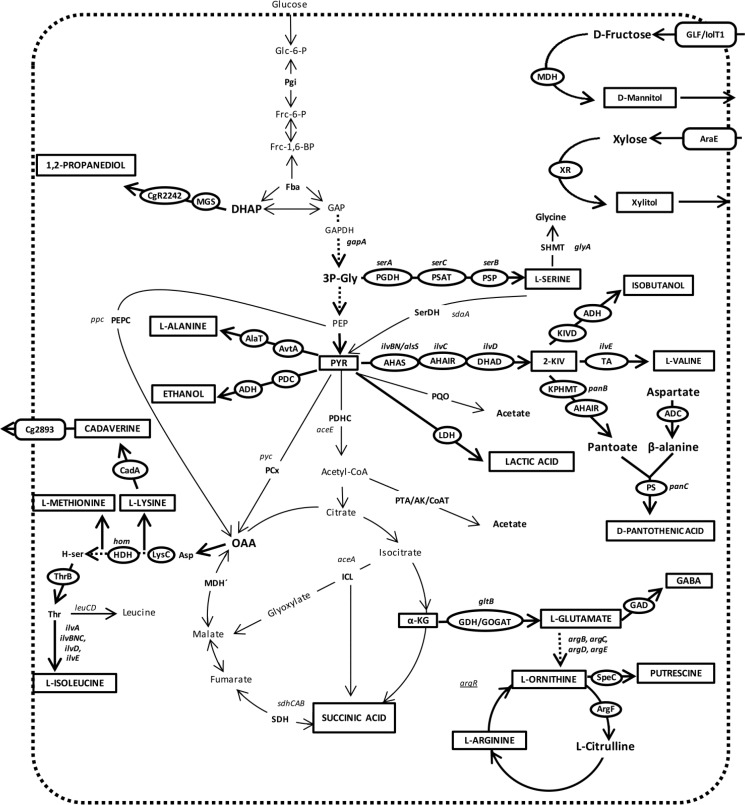
**Products for which *C. glutamicum* has been metabolically engineered**. The products are depicted in rectangles and the enzymes involved in the biosynthesis pathway (bold arrows) are shown in ovals. The gene names for which the designation is not the same as enzyme name are mentioned separately next to ovals. Broken arrows represent multiple steps. *argR* (underlined) is a gene-encoding regulator of the arginine pathway. Abbreviations: 2-KIV, 2-Ketoisovaleric acid; 3P-Gly, 3-phosphoglycerate; α-KG, α-Ketoglutaric acid; ADC, Aspartate decarboxylase; ADH, alcohol dehydrogenase; AHAIR, acetohydroxyacid isomeroreductase; AHAS, acetohydroxyacid synthase; AK, acetate kinase; AlaT, alanine aminotransferase; AraE, arabinose transporter; ArgF, L-ornithine carbamoyltransferase; Asp, aspartate, AvtA, valine-pyruvate aminotransferase; CadA, L-lysine decarboxylase; CoAT, acetyl-CoA:CoA transferase; DHAD, dihydroxyacid dehydratase; DHAP, dihydroxyacetonephosphate; Fba, fructose-bisphosphate aldolase; Frc-6-P, fructose-6-phosphate; Frc-1,6-BP, fructose-1,6-bisphosphate; GAD, glutamate decarboxylase; GAP, glyceraldehyde-3-phosphate; GAPDH, glyceraldehyde-3-phosphate dehydrogenase; GDH, glutamate dehydrogenase; Glc-6-P, glucose-6-phosphate; GLF, glucose/fructose facilitator; GOGAT, glutamate-2-oxoglutarate aminotransferase; HDH, homoserine dehydrogenase; H-ser, homoserine; ICL, isocitrate lyase; IolT1, inositol transporter; KIVD, 2-ketoacid decarboxylase; KPHMT, ketopantoate hydroxymethyltransferase; LDH, lactate dehydrogenase; LeuCD, 3-isopropylmalate dehydratase; LysC, aspartate kinase; MDH, mannitol dehydrogenase; MDH′, malate dehydrogenase; MGS, methylglyoxal synthase; OAA, Oxaloacetate; PCx, pyruvate carboxylase; PDC, pyruvate decarboxylase; PDHC, pyruvate dehydrogenase complex; PEP, phosphoenolpyruvate; PEPC, phosphoenolpyruvate carboxylase; PGDH, 3-phosphoglycerate dehydrogenase; PQO, pyruvate:quinone oxidoreductase; PSAT, phosphoserine aminotransferase; Pgi, glucose-6-phosphate isomerase; PS, pantothenate synsthase; PSP, phosphoserine phosphatase; PTA, phosphotransacetylase; PYR, pyruvate; SDH, succinate dehydrogenase; SerDH, serine dehydratase; SHMT, serine hydroxymethyltransferase; SpeC, ornithine decarboxylase; TA, transaminase; Thr, threonine; ThrB, homoserine kinase; XR, xylose reductase.

### III.1 Amino acids

#### III.1.1 L-valine

L-valine is an essential amino acid with application in feed and pharmaceutical industry and has a demand of several hundred tons per year [9]. Production of L-valine by *C. glutamicum* has been demonstrated both aerobically and anaerobically. Blombach et al. were able to engineer an aerobic L-valine producer by starting with a first-generation strain having an inactivated pyruvate dehydrogenase complex (PDHC) and overexpressing L-valine biosynthesis pathway genes *ilvBN*CE. In order to channel all pyruvic acid towards L-valine production, *pqo* encoding pyruvate:quinone oxidoreductase and *pyc* encoding pyruvate carboxylase were deleted. The former enzyme converts pyruvic acid to acetic acid and the latter converts it to oxaloacetic acid. Furthermore, to increase the flux via pentose phosphate pathway in order to meet the cofactor (NADPH) demand, pgi encoding phosphoglucose isomerase was also deleted (Fig. 2). The resulting strain produced 48 g l^-1^ L-valine in 90 hours in a growth decoupled manner during fed-batch fermentation [53].

For anaerobic production, Hasegawa et al. engineered a *C. glutamicum* strain by deletion of *ldh* encoding lactate dehydrogenase to prevent lactic acid formation and overexpression of a modified *ilvBNCD* (feedback resistant acetohydroxyacid synthase (encoded by *ilvN*) and a modified acetohydroxy acid isomeroreductase (encoded by *ilvC*) with preference for NADH instead of NADPH). Additionally, to allow use of NH3 instead of glutamate as amino donor, a Lysinibacillus sphaericus leucine dehydrogenase gene (*ilvE*) was expressed in *C. glutamicum* to catalyze the final transamination step. This approach yielded a *C. glutamicum* capable of producing up to almost 230 g l^-1^ L-valine in 48 hours under oxygen deprivation conditions [54].

#### III.1.2 L-isoleucine

L-isoleucine belongs to the aspartate family of amino acids and finds applications in food and pharmaceutical industry. In addition to being relatively long, L-isoleucine biosynthesis pathway shares genes with L-valine and L-leucine pathways and hence is subject to tight genetic and feedback regulation at multiple steps. Accordingly, these steps have been the targets of metabolic engineering in order to overcome the regulation and allow higher carbon flux towards L-isoleucine. An L-lysine producer was selected as a starting strain for L-isoleucine production because it has a high flux through the feedback resistant aspartate kinase (*lysC*^fbr^) reaction, which is the first step towards L-isoleucine formation from L-aspartate. Additionally *leuCD* was mutated which reduces formation of L-leucine as a byproduct. In this genetic background, chromosomal expression of three copies of *hom*^fbr^, a single copy of *ilvA*^fbr^ and overexpression of *ilvA*^fbr^ on a multiple copy vector yielded a strain that produced up to 21 g l^-1^ isoleucine via fed-batch fermentation [55]. *hom*^fbr^ encodes for a feedback-resistant homoserine dehydrogenase that catalyzes homoserine formation and its overexpression increases flux towards threonine, which is the precursor amino acid of L-isoleucine. *ilvA*^fbr^ encodes for a feedback-resistant threonine dehydratase that catalyzes the first step in conversion of L-threonine to L-isoleucine. Upon investigation of another L-isoleucine producer it was also shown that L-isoleucine export, rather than precursor supply or enzyme activities, is the limiting factor in obtaining higher titers and therefore presents a prospective opportunity for further improvement.

#### III.1.3 L-serine

L-serine finds applications in pharmaceutical and cosmetic industry and is produced in the range of 300 tons per year [56]. It is degraded by *C. glutamicum* and holds a key position in central metabolism. Hence, mere overexpression of serine biosynthetic pathway genes *serA*^▵197^ (made insensitive to feed-back regulation by truncation of 197 amino acids at C-terminus), serC and serB was not enough for serine production. *serA*^▵197^ encodes for 3-phosphoglycerate dehydrogenase (PGDH) [57] that catalyzes the first step of serine formation by converting the glycolysis intermediate 3-phosphoglycerate to hydroxypyruvate phosphate. *serC* and *serB* encode phosphoserine aminotransferase (PSAT) and phosphoserine phosphatase (PSP) respectively that are responsible for the next two steps resulting in L-serine formation. [56, 58]. In addition to the overexpression of these L-serine biosynthesis pathway genes, a disruption of serine hydroxymethyltransferase gene (*glyA*) and serine dehydratase gene (*sdaA*), whose products are responsible for L-serine conversion to glycine and pyruvic acid respectively, was essential to form L-serine (9 g l^-1^) as a product [56, 58].

#### III.1.4 L-arginine

L-arginine is the most abundant nitrogen carrier in animals and is essential for ammonia detoxification. Its clinical application in treatment of a variety of diseases including diabetes, AIDS and heart diseases has been reported [59]. Using reverse engineering, Ikeda et al., combined mutations identified in classical producers to engineer an L-arginine and L-citrulline producer. These mutations were found to be in the arginine repressor gene *argR* and in the *argB* gene whose product catalyzes the N-acetylglutamate kinase reaction in the beginning part of the arginine biosynthesis pathway [60]. Accordingly, *argR* was deleted and the two amino acid substitutions observed in classical producer (A26V and M31V) were introduced in the wild type N-acetylglutamate kinase resulting in higher productivity of L-arginine and L-citrulline at the suboptimal temperature of 38°C. However, this strain could not produce L-arginine at the optimal temperature of 30°C. Later, Schneider et al., [35] starting with a similar strain carrying *argR* deletion and overexpressing a feedback resistant *argB*, created *C. glutamicum* ARG1 which led to production of about 7 g l^-1^ L-arginine at 30^°C^. Overexpression of the arabinose utilization operon *araBAD* (Fig. 1) in ARG1 allowed it to utilize arabinose or a mixture of glucose and arabinose as carbon source, resulting in production of about 8.3 g l^-1^ L-arginine, although complete utilization of arabinose was not possible [35].

#### III.1.5 L-methionine

L-methionine belongs to the aspartate family of amino acids and is the third most-produced amino acid (tons per annum), next to L-glutamate and L-lysine. However, so far, its fermentative production has not been proven to be industrially feasible. This is probably because with a requirement of 7 mol ATP and 8 mol NADPH per mol L-methionine, it is the most demanding amino acid for fermentative production. By deletion of a gene producing the competing amino acid L-threonine (*thrB*) and by deregulating feedback inhibition of homoserine dehydrogenase (*hom*), Park et al, engineered a L-lysine producing *C. glutamicum* strain for production of 2.9 g l^-1^ L-methionine [61]. However, depletion of glucose in the medium led to disappearance of the produced L-methionine. Although not reflected in academic literature, commercial methionine fermentation could be a reality in the near future.

#### III.1.6 L-ornithine

L-ornithine is a non-proteinogenic amino acid that is an intermediate of the arginine biosynthesis pathway and might be used for treating liver diseases [62]. It was shown that deletion of *argF* gene encoding L-ornithine carbamoyltransferase to stop L-ornithine conversion and of *argR* encoding repressor of arginine biosynthesis pathway were sufficient for L-ornithine formation in *C. glutamicum* in milligrams per liter range [63]. Further optimization for L-arginine requirement of strains and introduction of arabinose utilization pathway (Fig. 1) resulted in L-ornithine titers of up to almost 26 g l^-1^ in 72 hours using glucose and arabinose as a carbon source [35].

#### III.1.7 γ-Aminobutyric acid

γ-Aminobutyric acid (GABA) is a non-proteinogenic amino acid that functions as the main inhibitory neurotransmitter of the mammalian nervous system and has pharmaceutical relevance for use as anti-anxiety agent and in tranquilizers, diuretics and analgesics. It can be formed from glutamate via a single decarboxylation step and hence *C. glutamicum*, being a natural L-glutamate producer, can serve as a competitive production platform. Using heterologous expression of *Lactobacillus brevis* genes for: i) glutamate decarboxylase (*gadB2*) to catalyze glutamate decarboxylation to GABA, ii) L-glutamate/GABA antiporter gene *gadC* and iii) the transcriptional regulator gene *gadR*, Shi and Li constructed a strain that could produce more than 2 g l^-1^ GABA in 72 hours [64]. Zhao et al. reported the GABA transporter in *C. glutamicum* responsible for its uptake and were able to show that disruption of its gene led to increased GABA production [65]. Production of GABA using *C. glutamicum* has the advantage that it is independent of external glutamate supply and complex media ingredients, which is the case when using lactic-acid bacteria (LAB) for its production. However the GABA production levels are higher using LAB and hence there is further need for improvement of *C. glutamicum* strains.

#### III.1.8 D-amino acids

Production of D-amino acids was also shown to be possible in *C. glutamicum* whereas other bacteria such as *E. coli* are sensitive to their presence even in low concentrations. Heterologous expression of *Pseudomonas taetrolens* racemase (*argR*) resulted in extracellular accumulation of equimolar amounts of L- and D- enantiomers of arginine, ornithine or lysine whereas D-serine concentration exceeded that of L-serine [66]. D-amino acids are used as building blocks in chemistry and hence their production via fermentation is of interest.

### III.2 Diamines

#### III.2.1 Putrescine

Putrescine is a biogenic diamine that serves as a monomer in the production of polyamides e.g. nylons-4,6. Polyamides are produced on a multi-million-ton scale per annum predominantly from petrochemical routes and currently there is an increasing interest in their fermentative production. Putrescine can be formed via arginine-or ornithine decarboxylase pathways and it was shown that the latter results in better production levels [67]. The genetic engineering involved deletion of *argF* and *argR* in order to channel ornithine (putrescine precursor) solely towards putrescine and to relieve genetic repression of the arginine biosynthesis pathway respectively. Heterologous expression of the *E. coli* ornithine decarboxylase gene (speC) then led to formation of up to 6 g l^-1^ putrescine in 60 hours in a growth independent manner when supplemented with arginine [67]. In the next step, introduction of a plasmid addiction system expressing *argF* resulted in both relief of arginine auxotrophy and growth dependent putrescine production [68].

#### III.2.2 Cadaverine

Like putrescine, cadaverine is also a diamine, which can be used for polyamide production [69]. Mimitsuka et al. were the first to engineer *C. glutamicum* for cadaverine production by replacing *hom* with the L-lysine decarboxylase gene cadA from E. coli, resulting in production of 2.6 g l^-1^ cadaverine from glucose in 18 hours [70]. *hom* deletion functioned to stop carbon flow downstream to the formation of branched chain amino acids (L-valine, L-leucine, L-isoleucine) and diverted it instead towards cadaverine formation via lysine. In separate studies with different strains, expression of *xylA* and *xylB* that encode xylose utilization enzymes (Fig. 1) [37] and of amyA that encodes starch hydrolysis enzyme, (Fig. 1) [30] allowed cadaverine production from these substrates. Later on, overexpression of a putative permease, encoded by cg2893 [37] and deletion of a putative diaminopentane acetyltransferase, encoded by NCgl1469 (cg1722) [37] were also shown to result in higher product formation and elimination of acetylated byproduct respectively.

### III.3 Organic acids

#### III.3.1 Succinic acid

Succinic acid can serve as a platform chemical and hence there is plentiful research going on to produce it via fermentation [45, 71–74]. The annual demand for the free acid is 15,000 tons whereas its salts are used annually in amounts up to 92,000 tons as deicers. For a feasible industrial process, the target succinic acid amount and productivity have been set at 150 g l^-1^ and 5 g l^-1^ h^-1^ respectively [75]. By deleting lactate dehydrogenase gene (*ldhA*) and overexpressing the anaplerotic pyruvate carboxylase gene (*pyc*), a *C. glutamicum* strain was made that produced 146 g l^-1^ succinic acid with a productivity of 3.2 g l^-1^ h^-1^ under anaerobic conditions [73]. The former modification avoided lactic acid as byproduct and the latter drives the carbon flux in to the reductive tricarboxylic acid (TCA) cycle. In a pioneering study redox-balanced anaerobic succinate production could be achieved [72]. Besides the aforementioned modifications (overexpressing a modified *pyc*^P458S^ instead of wild type *pyc*) and deletion of all known acetic acid forming genes (*cat, pqo, ackA-pta*), overexpression of the glyceraldehyde 3-phosphate dehydrogenase (*gapA*) gene led to significantly reduced acetic acid (byproduct) formation, accelerated glucose consumption and very high succinic acid titers. Heterologous production of *Mycobacterium vaccae* formate dehydrogenase (*fdh*) entailed use of formate as cosubstrate [72]. Thus, coutilization of glucose with formate allowed for redox-balanced anaerobic succinate production by this recombinant strain.

Under aerobic conditions combined overexpression of phosphoenolpyruvate carboxylase (*ppc*) and pyruvate carboxylase (*pyc*) genes – to propel anaplerosis – and deletion of acetic acid forming genes (see above) and of succinic acid oxidizing genes (*sdhCAB*) – to prevent succinic acid conversion – resulted in a strain producing 10.6 g l^-1^ succinic acid in a growth decoupled manner. This is the highest titer reported for aerobic succinic acid production in *C. glutamicum* [74]. Subsequent overexpression of glycerol utilization genes *glpFKD* (Fig. 1) allowed this strain to produce almost the same amount of succinic acid from glycerol [45].

#### III.3.2 Pyruvic acid

Pyruvic acid finds applications in many industries including food, cosmetic and pharmaceutical. Production costs for chemical synthesis of pyruvate of US$ 8000-9000/ton have been reported [76]. Hence, there is a lot of interest in its fermentative production. It also holds an important position in the central carbon metabolism and serves as a connecting point between glycolysis and TCA cycle. Additionally, it is a branching point for the formation of a variety of organic and amino acids. Consequently, the approach for pyruvic acid engineering focused on attenuation of these ‘branches’ to allow for its accumulation. Acetyl-CoA, acetic acid, lactic acid and alanine production were minimized by deletion of pyruvate dehydrogenase (*aceE*), pyruvate:quinone oxidoreductase (*pqo*), lactate dehydrogenase (*ldh*) and of alanine aminotransferase (*alaT*) plus valine-pyruvate aminotransferase (*avtA*) genes respectively. Additionally, the C-terminus of acetohydroxyacid synthase (AHAS) was deleted (▵C-T *ilvN*) to prevent overflow metabolism into L-valine synthesis. Titers of more than 45 g l^-1^ were achieved using this strain [77].

#### III.3.3 Lactic acid

Lactic acid is used in cosmetic and pharmaceutical industry. Its major market, however, is packaging where polymers of lactic acid are in demand. *C. glutamicum* wild type was shown to produce L-lactic acid under oxygen deprivation conditions and in fed-batch fermentation could produce 52 g l^-1^ L-lactic acid in 8 hours. In a continuous process using cell-recycling L-lactic acid production continued for 360 hours [78]. Succinic acid and acetic acid were also produced but only in lower amounts.

A drawback associated with poly L-lactic acid (PLLA) is its low melting point. One way to improve this property is production of a stereocomplex of PLLA and Poly D-lactic acid. To engineer a strain that can produce D-lactic acid a *L. delbrueckii* D-lactate dehydrogenase gene was expressed in *C. glutamicum* with a deletion of the lactate dehydrogenase (*ldhA*) gene. The resulting strain produced 120 g l^-1^ of 99.9% optically pure D-lactic acid in less than 30 hours [79].

#### III.3.4 α-Ketoglutaric acid

Like succinic acid, α-ketoglutaric acid is a TCA cycle intermediate and is in demand by pharmaceutical and food industry. Jo et al. engineered an α-ketoglutaric acid producer by starting with a L-glutamate producing strain as it already has a high α-ketoglutarate availability. Glutamate dehydrogenase and glutamate-2-oxoglutarate aminotransferase (GOGAT) encoding genes, *gdh* and *gltB*, respectively, were disrupted to abolish conversion of α-ketoglutaric acid to L-glutamate. In order to channel the precursor isocitrate solely to α-ketoglutaric acid, *aceA* encoding isocitrate lyase that converts isocitric acid into glyoxylic acid and succinic acid was also disrupted. The resulting triple mutant was able to accumulate 12.4 g l^-1^ α-ketoglutaric acid after 72 hours [80].

#### III.3.5 2-Ketoisovaleric acid

2-Ketoisovaleric acid (2-KIV) is used by pharmaceutical industry in drugs for treating kidney patients. It is a precursor of the amino acid L-valine and is formed from pyruvic acid in three reactions catalyzed by acetohydroxyacid synthase (AHAS, encoded by *ilvBN*), acetohydroxyacid isomeroreductase (AHAIR, encoded by *ilvC*) and dihydroxyacid dehydratase (DHAD encoded by *ilvD*). Accordingly, the engineering approach adopted by Krause et al. [81]involved overexpression of these biosynthetic pathway genes (*ilvBNCD*) in combination with deletion of: i) *ilvE* to prevent L-valine formation ii) *aceE* to channel pyruvate to 2-KIV formation and to allow growth decoupled production and iii) *pqo* to further enhance carbon flow to 2-KIV synthesis. This resulted in the growth-decoupled formation of approximately 22 g l^-1^ 2-KIV by the engineered *C. glutamicum* strain [81].

#### III.3.6 D-Pantothenic acid

D-Pantothenic acid (Vitamin B5) can be synthesized by microorganisms and plants but not by animals and is therefore demanded as nutritional requirement for livestock and humans. Its worldwide market is reported to be 10,000 tons/year [82]. Starting from 2-ketoisovaleric acid, pantothenic acid can be synthesized in four enzymatic steps. The enzymatic reactions of the pantothenic acid biosynthesis pathway (starting from pyruvic acid) are also partly shared by the L-isoleucine and L-valine biosynthesis pathways. Its production was elucidated in a first-generation *C. glutamicum* production strain by overexpression of ketoisovaleric acid pathway genes (*ilvBNCD*) and of D-pantothenic acid biosynthesis pathway genes (*panBC*). To prevent L-isoleucine formation, *ilvA* was deleted [83]. This strain accumulated up to 1 g l^-1^ D-pantothenic acid. Metabolic analysis of this strain identified a high flux of the precursor 2-ketoisovaleric acid to L-valine instead of D-pantothenic acid as the main limitation for pantothenic acid production [84]. Using this information, a promoter down mutation was introduced in the first generation strain to attenuate *ilvE* expression and hence to stop L-valine formation. A duplication of *panBC* expression to increase flux into D-pantothenic acid formation was also done. This second-generation strain was able to attain titers of 1.75 g l^-1^ [85]. The precursor and other byproducts from associated pathways were still secreted by this strain indicating an opportunity for further metabolic engineering.

### III.4 Alcohols

#### III.4.1 Ethanol

Ethanol fermentation for use as fuel has a well-known history and ethanol production in USA alone was more than 13 billion gallons in 2010 [86]. Ethanol production was achieved in *C. glutamicum* by heterologous expression of the *Zymomonas mobilis* pyruvate decarboxylase gene (*pdc*) and overexpression of an alcohol dehydrogenase gene (*adhB*). The former enzyme catalyzes decarboxylation of pyruvic acid to acetaldehyde, which is in turn reduced by the latter enzyme to ethanol. Ethanol production was observed with these modifications but lactic acid and succinic acid were also produced as byproducts. Disruption of the lactate dehydrogenase (*ldh*) and phosphoenolpyruvate carboxylase (*ppc*) genes relieved this problem and the resulting strain produced about 10 g l^-1^ ethanol under oxygen deprivation conditions [87]. Interestingly, *C. glutamicum* can well resist various growth inhibitors such as furans, phenols and acids that are formed during lignocellulose pretreatment under the chosen conditions, thereby opening up the prospect of ethanol production from biomass [88].

#### III.4.2 Isobutanol

Recently, the research interest has shifted from ethanol fermentation to production of higher alcohols for use as biofuel due to various advantages offered by the latter such as higher energy density, compatibility with existing infrastructure and lower corrosivity, vapor pressure and hygroscopicity. Isobutanol is one such higher alcohol and can be produced from 2-KIV in two steps. Because *C. glutamicum* has been successfully engineered for production of the isobutanol precursor 2-KIV [81] it appeared as a suitable organism for isobutanol production. Smith et al. engineered a producer strain by heterologous expression of a *B. subtilis alsS*, whose product (AHAS) catalyzes the conversion of pyruvic acid to acetolactate, in combination with the overexpression of *ilvC* and *ilvD* whose products in turn catalyze the next two steps towards 2-KIV formation. Heterologous expression of a *Lactococcus lactis* 2-ketoacid decarboxylase gene (*kivd*) and overexpression of native alcohol dehydrogenase gene (*adhA*) then converted 2-KIV to isobutanol. *pyc* encoding pyruvate carboxylase (PCx) and *ldh* were deleted to direct more carbon through the isobutanol pathway. The resultant strain produced 4.9 g l^-1^ isobutanol in 120 hours [89]. Blombach et al achieved more than twofold higher titers after re-engineering their 2-KIV producing strain for isobutanol biosynthesis [90]. Apart from deleting *ldh*, they also deleted a number of other genes to further increase pyruvic acid, 2-KIV and cofactor availability: *aceE* and *pqo* deletions served the first purpose, *ilvE* deletion the second and *mdh* deletion the third and last. However, the resultant strain grew very poorly on glucose. To overcome this drawback and to improve redox balance, *E. coli pntAB* operon encoding transhydrogenase was additionally expressed. This strain could then produce 13 g l^-1^ isobutanol under oxygen deprivation conditions [90].

#### III.4.3 1,2-propanediol

1,2-propanediol holds a worldwide market of more than half million tons per annum and is used in food, cosmetic and pharmaceutical industries. Production of 1,2-propanediol by *C. glutamicum* was observed after heterologous expression of *E. coli* methylglyoxal synthase gene (*mgs*) and overexpression of a putative aldo-keto reductase gene (*cgR_2242*). The former enzyme catalyzes the conversion of the glycolytic intermediate dihydroxyacetone phosphate (DHAP) to methylglyoxal, which is reduced to 1,2-propanediol by the latter enzyme. This approach resulted in a strain with a production capability of 1.8 g l^-1^ in 90 hours via fed-batch fermentation [91]. A high concentration of acetol, a 1,2-propanediol precursor, was also produced and presents a potential hurdle to reach higher 1,2-propanediol titers.

### III.5 Sugar alcohols

#### III.5.1 D-Mannitol

D-Mannitol is used as an osmotic diuretic in pharmaceutical industry and as an alternative sweetener for diabetic patients in food industry and is produced using a chemical hydrogenation procedure. D-Mannitol production from D-fructose as substrate was demonstrated using resting cells of *C. glutamicum* [92]. The metabolic engineering steps included heterologous expression of: i) *Leuconostoc pseudomesenteroides mdh*, encoding a mannitol dehydrogenase, that catalyzes D-fructose to D-mannitol conversion, ii) *Mycobacterium vaccae* N10 *fdh*, encoding a formate dehydrogenase, that ensures ample cofactor supply in the presence of formate and iii) *Z. mobilis* glucose/fructose facilitator *glf* whose product enhances direct D-fructose uptake. The strain was able to form 285 g l^-1^ D-mannitol over a period of 96 hours using a fed-batch approach [92]. Later on, the native transporters of *C. glutamicum* for D-fructose were identified and it was shown that overexpression of one of these (*iolT1*), instead of heterologous expression of *glf*, also resulted in increased D-fructose uptake rate [93].

#### III.5.2 Xylitol

Xylitol finds relevance in food industry where it is used as a dietetic sucrose alternative because it has one third the calories of sucrose while being equally sweet and can be produced directly from xylose via a single reduction step. The metabolic engineering for creating a *C. glutamicum* xylitol producer included: i) heterologous expression of a *C. glutamicum* ATCC31831 pentose transporter gene (*araE*), inserted into the chromosomal DNA to improve direct xylose uptake, ii) heterologous expression of a *Candida tenuis* gene encoding a single-site mutated xylose reductase (XR) to catalyze xylitol formation, iii) deletion of the lactate dehydrogenase gene (*ldh*) to allow use of cofactor utilized in lactic acid formation reaction for xylitol production instead, and iv) deletion of phosphoenolpyruvate-dependent fructose phosphotransferase gene (*ptsF*) and xylulokinase gene (*xylB*) (Fig. 1) to prevent formation of xylitol-phosphate which is toxic for cells. The former enzyme catalyzes xylitol uptake via the phosphoenolpyruvate-dependent fructose phosphotransferase system and the latter is probably involved in its phosphorylation to yield the toxic intermediate. Indeed the deletion of these genes resulted in insensitivity to xylitol and enhanced xylitol productivity. The strain was shown to produce 166 g l^-1^ xylitol in 21 hours using resting cells and a fed-batch approach [94].

### III.6 Cofactor regeneration

Cofactor availability is critical for biotransformation reactions as mentioned above for sugar alcohols. It has recently been shown that deletion of either 6-phosphofructokinase gene (*pfkA*) or glyceraldehyde 3-phosphate dehydrogenase gene (*gapA*) resulted in a higher NADPH per glucose yield as compared to wild-type strain, which could then be used for reductive biotransformation of the prochiral methyl acetoacetate to the chiral (*R*)-methyl 3-hydroxybutyrate (MHB) via expression of a *Lactobacillus brevis* alcohol dehydrogenase. With this approach, an almost three fold increase of MHB yield could be observed. Deletion of these genes halts the flow through glycolysis, diverts it to pentose phosphate pathway (PPP) and generates a semi-cyclic (*pfkA* deletion) or cyclic (*gapA* deletion) PPP, resulting in an increased cofactor production and availability [95].

## IV. Summary and Outlook

It is evident that currently there is plentiful research going on to metabolically engineer *C. glutamicum*: production of more than two-third of the products discussed in this review has been made possible in the last four years only. The availability of the genome sequence and genetic engineering tools for this organism make its rapid and rational manipulation possible. So far, ‘omics’ technologies have not been extensively used in context of metabolic engineering of this bacterium, whereas they do hold the potential to elucidate production mechanisms and constraints on a systems-wide level. It can be expected that metabolic engineering of *C. glutamicum* will benefit from the increasing availability of gene sequences for pathway enzymes from many different organisms (not only *E. coli* and *S. cerevisiae*). In many cases, however, biochemical data rather than only a gene sequence are required to guide metabolic engineering and it is likely that more resources need to be directed towards physiological and biochemical experimental work. It is expected that pathway modeling, ideally in the context of a genome-scale metabolic model will contribute to metabolic engineering. Moreover, technical and conceptual advances to gradually control expression of not one or two, but rather tens of genes will be required to install larger synthetic pathways.

*C. glutamicum* is an attractive candidate for metabolic engineering as the biotechnological industry looks back at more than fifty years of safe production in the food and feed industry with this bacterium. Moreover, annual million-ton-scale production, such as of L-lysine and L-glutamate by *C. glutamicum*, cannot be overlooked. The examples described here demonstrate that *C. glutamicum* holds the potential as a general microbial production platform.
